# Mortality in the Army and Navy

**Published:** 1847-02

**Authors:** 


*Mortality in the Army and Navy.*—The numbers of those who perish in battle, or afterwards from wounds, is small compared to those who die from other causes. During the last three years of the Peninsular war, the total number of deaths in the British Army, amounted annually to about 16 per cent. of the whole force. Of these only 4 per cent. died in battle or of wounds which proved fatal soon after. The number of men sick in hospitals usually averaged about one-fourth of the whole. In less than three years and a half out of a force the average strength of which was 61,500 men, nearly 34,000 died, and of these only one-fourth fell by the sword; and this enormous mortality occurred among a body of men, all of whom, a short time previously, must have been in the healthiest vigor of youth or prime of manhood; so that it required the annual sacrifice of 6,400 able-bodied men, to keep in the field a working force of less than 50,000 men! If such was the amount of suffering and waste of life, when every expedient was adopted that foresight could suggest to provide proper food and raiment, and every other attainable comfort both in sickness and in health, what must it be when these precautions are neglected? Of such neglect and its terrible and execrable consequences, Napoleon's campaigns of 1812 and 1813 afforded memorable examples.—From want of proper supplies alone, the French troops perished literally by hundreds of thousands!

There is a remarkable difference between our land and sea services. The channel fleet which consisted of twenty-four sail of the line with frigates, &c., on its return to Torbay in September, 1800, after a four months' cruise, sent only sixteen men to hospital. The average mortality in the years 1810, 1811, and 1812, was only 3½ per cent.; since 1830 it has not been more than 1·4 per cent., which is less than the general average among of men of the same age on shore.—*London Med. Gaz.—Med. News.*

*Therapeutic action of Chloride of Sodium* (*common salt*).—M. Plouviers, of Lille, stated to the Academy of Sciences, that since 1842, he has been experimenting with this article. He found that after taking during six weeks, a teaspoonful, then a teaspoonful and a half of this salt every morning in a cup of milk, he became stronger, more active and weighed 5 kilogrammes (about 15 pounds) more than he did before. In continuing the use of the salt, he became plethoric, and had to cease taking it. Subsequent experience multiplied and varied, has convinced him that common salt possesses a high importance in assisting digestion. He thinks in persons of sanguine temperament or apoplectic tendency it is dangerous; but to weak constitutions without disease, it is incontestably useful; and to laborers and the poor, it would assist much in their nourishment.—*Gaz. Méd.—Southern Med. and Surgical Journal.*

